# Staying True to the Core of Public Health Science in Times of Change

**DOI:** 10.3389/fpubh.2021.653797

**Published:** 2021-05-21

**Authors:** Kristina Areskoug Josefsson, Alexandra Krettek

**Affiliations:** ^1^Faculty of Health Sciences, VID Specialized University, Sandnes, Norway; ^2^The Jönköping Academy for Improvement of Health and Welfare, Jönköping University, Jönköping, Sweden; ^3^Department of Behavioural Sciences, Faculty of Health Sciences, Oslo Metropolitan University, Oslo, Norway; ^4^Department of Public Health, School of Health Sciences, University of Skövde, Skövde, Sweden; ^5^Department of Internal Medicine and Clinical Nutrition, Institute of Medicine, Sahlgrenska Academy at University of Gothenburg, Gothenburg, Sweden; ^6^Department of Community Medicine, Faculty of Health Sciences, UiT The Arctic University of Norway, Tromsø, Norway

**Keywords:** public health, digital health, transdisciplinary, methods, scientific core

## Introduction

We currently experience rapid technological development and societal changes. Most notably, health care is transformed with preventive medicine and public health efforts being driven by use of new digital health technologies. This leads to an unexpected development for Public Health Science, the discipline that provides the scientific platform for efforts focused on the health of the public. The traditional core of its discipline is challenged by new approaches, such as extensive use of digitalization in various ways, to promote and maintain health.

### The Current Challenge for Public Health Science in Modern Society

As core areas within Public Health Science are given less attention or are entirely removed for the benefit of digital content—education in Public Health Science is left at risk of becoming limited. Indeed, the Association for Schools of Public Health in the European Region outlines extensive generic core competences in Public Health Science for the Public Health Professional (1). These include quantitative and qualitative methods, determinants of health, population health and its material and environmental determinants, health policy issues, organizational theory and management as well as aspects of health promotion and disease prevention, and last but not least; ethical aspects (1).

The development of digital technologies, together with advancements in artificial intelligence, is the most significant on-going transformation of contemporary society which also influences developments in the public sector (2). Indeed, in this context, it is vital to maintain a neutral stance toward digitalization—it is not without its own risks. A critical perspective is needed when exploring the potential of new technologies while at the same time keeping in mind any potential unintended consequences. There are also issues such as sociocultural disadvantages and marginalization of groups with power relations speaking for the interests of some groups and organizations while others are ignored (3). Indeed, even during the ongoing pandemic, digital innovations have been applied in an attempt to efficiently respond to COVID-19. For this particular context, there are multiple barriers that hamper efficient implementation. These include legal, ethical and privacy issues as well as constraints related to workforce and organizational factors (4).

### Knowledge of Traditional Areas of Public Health Science Is Needed

At the same time, traditional public health knowledge, especially within the areas of epidemiology and infectious disease control, are critically needed during health emergencies like the ongoing COVID-19 pandemic. The force of change is constantly challenging Public Health Science to evolve (5) and impels us to make choices on how to successfully address those changes. This clearly shows the need for in depth-knowledge of traditional Public Health Science and its scientific perspectives. Public Health Science provides methods to understand effects of public health interventions on all levels. It thus creates important strategies to promote and sustain health for all—both now, and, most importantly, in the future.

From our professional perspectives, we see that there is a danger of losing the core of Public Health Science in the ongoing digitalization trend. Digital tools are sometimes proposed as being a novel part of the core of Public Health Science—instead of being just tools. This could be due to the omnipresent influence from external actors challenging the knowledge characteristics of Public Health Science. We find it crucial to keep this potential danger in focus.

### Public Health Science—A Multidisciplinary Umbrella With Extensive Methodological Toolbox

**Figure 1:**
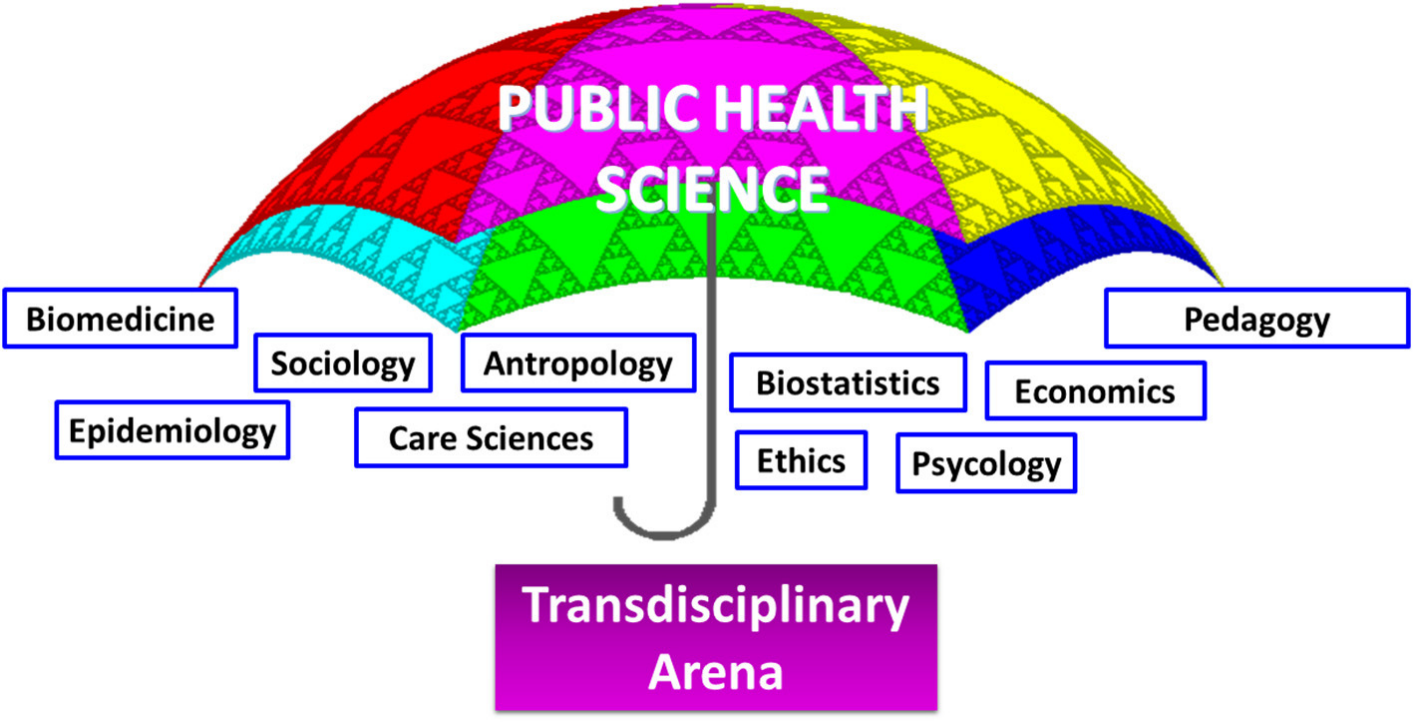
Public Health Science can be seen as a health umbrella with several scientific areas feeding relevant parts of their knowledge and methodological tools into the discipline, thereby contributing to a transdisciplinary arena.

Public Health Science can be seen as a health umbrella with several scientific areas feeding relevant parts of their knowledge and research methodologies into the discipline, thus creating its transdisciplinary foundation (Figure 1). Public Health Science focuses on promoting health, preventing disease as well as prolonging and improving quality of life primarily on the population level (6), but indirectly encompasses all levels i.e., individual, societal and global. An overview of the extent of Public Health Science as a discipline is described elsewhere (7). The definition proposed by Acheson, and used by the WHO European Region, describes that Public Health Science is: “the science and art of preventing disease, prolonging life and promoting health through the organized efforts of society” (8). This is achieved through collective action of many stakeholders in both the private and the public sector (9).

Indeed, as Public Health Science is a transdisciplinary arena for research and teaching, a methodological width in theories, concepts, approaches and practices is needed. Another necessity is the understanding of what each individual scientific area can contribute with. For Public Health Science, there is great strength in having access to, and proficiency in, a vast variety of scientific methods (10). Public Health Science has employed research methods from different areas since its conception in the 1880s. Indeed, transdisciplinary and interdisciplinary approaches are a must for successfully addressing global health issues (11). However, Public Health Science is not distinguished by, and should not primarily be associated with, its large methodological toolbox.

### The Core of Public Health Science

The core encompasses the three main pillars of health promotion, disease prevention and environmental health. We consider that the three pillars are in themselves influenced by health management on the micro-meso-macro levels in health and welfare systems.

However, Public Health Science can never be static. It needs to keep a strong focus on its core values of focusing on the health of the population and use all possibilities to promote health for all. In fact, the primary focus of Public Health Science is to maintain health, rather than focusing on only clinical ways of disease prevention, care and cure. However, it should be noted that there is no distinct boundary and overlapping areas do occur, e.g., between health promotive and disease preventive efforts.

As educators and researchers in Public Health, we consider it essential to hold on to the scientific core of Public Health Science when teaching and mentoring junior researchers within this field. In addition, maintaining a clear core of Public Health Science may help both the general public and the media in understanding the value of Public Health Science as an independent discipline. This is important as Public Health Science is often confused with clinical medicine and treatment of disease and also directly translated to public health implying “the health of the public.” Such aspects endanger Public Health Science as an independent discipline.

## Discussion

### Staying True to the Core of Public Health Science—But Adding Digital Tools

We strongly advocate for keeping the core of Public Health Science, where diversity is a strength of the discipline. Increased possibilities with digitalization within public health measures are important additions to the toolbox of Public Health Science (12) as the discipline needs to adapt to the everyday reality of the world and concurrently adopt new tools. Indeed, digital innovation is at the core of Nordic healthcare and helps combat global healthcare challenges (13). But such methods should be seen only as an addition and not be allowed to transform the core of Public Health Science as such.

In fact, there is also a great challenge in the ongoing digital transformation, as such tools may even negatively impact health (14). One example is the possibility to be constantly online on various social media and health measurement apps. This may lead to stress and worry instead of the intended positive effects. Another issue is the emerging availability of digital medical consultations. While these are easily accessible and are a positive development for some population groups, they may also lead to unnecessary health care consumption and increasing costs. The costs of digital services may lead to less available funding for traditional non-digital health care.

When making changes in health and welfare services, there is a risk of not meeting the needs for those not being able to use digital services. The WHO released the first guidelines in 2019 for digital health interventions (15). These guidelines have recently been reviewed in the context of the current research situation (16). Along the same line, in a recent statement the European Public Health Alliance and the European Public Health Association address the importance of a more people-focused approach when applying digital tools to health and care (17). They also stress that such tools must be accessible, affordable, inclusive and transparent. Critically addressing both intended and unintended effects of such tools is therefore vital (17).

## Conclusion: The Way Forward

Having said this, we strongly support the idea of a “Beautiful Marriage” between the worlds of Digital Health and Public Health Science as proposed by Jakab (18), while at the same time keeping the core of Public Health Science intact. Thus, not limiting Public Health Science to use of digital tools, but encompassing digitalization together with other scientific methods in Public Health Science. The current digitalization provides exciting possibilities, with constant development of new technologies which in turn present even more possibilities. The widespread use of mobile phones globally provides opportunities for digital users to collect health data at fast speed, thus engaging in co-productive Public Health research. Hence, digitalization can enrich research within Public Health Science by presenting larger opportunities for individuals and groups to actively partake in such research, instead of individuals being studied by others.

Active collection of health data via apps and self-tracking devices can provide a treasure chest of big data of health behaviors for researchers in Public Health Science, as well as companies interested in using such data for profitable purposes (14). In addition, digital tools enhance the ability for easy access to updated health information for diverse population groups. This can be clearly seen during the ongoing COVID-19 pandemic, where news apps provide information in several languages that also consider the health literacy level of the target population. However, such a strategy also includes a risk of fake information being spread just as quickly and easily, which is a serious threat to the health of the public (14). As one of the advantages of digitalization is the potential for increased health equity, researchers and educators within Public Health Science should actively engage to promote the use of digital tools for such purpose while still emphasizing that digitalization is a tool and not the goal or part of the core of Public Health Science.

## Author Contributions

All authors listed have made a substantial, direct and intellectual contribution to the work, and approved it for publication.

## Conflict of Interest

The authors declare that the research was conducted in the absence of any commercial or financial relationships that could be construed as a potential conflict of interest.
